# Cost-effectiveness of apixaban vs. aspirin for the reduction of thrombo-embolism in high-risk patients with device-detected atrial fibrillation: insights from the ARTESiA trial

**DOI:** 10.1093/europace/euaf195

**Published:** 2025-08-31

**Authors:** Andre Lamy, Roopinder K Sandhu, Wesley Tong, William F McIntyre, Renato D Lopes, Christopher B Granger, David J Wright, Jens C Nielsen, Valentina Kutyifa, Julia W Erath, Marco Alings, David H Birnie, Dan Atar, Stefan H Hohnloser, Cecilia Linde, Josef Kautzner, Juan Benezet-Mazuecos, A John Camm, Christian Sticherling, Michael R Gold, Charlotte E Larroudé, Jeff S Healey

**Affiliations:** Population Health Research Institute, McMaster University, 237 Barton Street East, Hamilton, Ontario, L8L2X2, Canada; Canadian VIGOUR Centre, University of Alberta, Edmonton, AB, Canada; Department of Cardiac Sciences, University of Calgary, Calgary, AB, Canada; Population Health Research Institute, McMaster University, 237 Barton Street East, Hamilton, Ontario, L8L2X2, Canada; Population Health Research Institute, McMaster University, 237 Barton Street East, Hamilton, Ontario, L8L2X2, Canada; Duke Clinical Research Institute, Duke University, Durham, NC, USA; Duke Clinical Research Institute, Duke University, Durham, NC, USA; Department of Cardiology, Liverpool Heart and Chest Hospital, Liverpool, UK; Department of Clinical Medicine, Aarhus University, Aarhus, Denmark; Department of Cardiology, Aarhus University, Aarhus, Denmark; Heart and Vascular Center, Semmelweis University, Budapest, Hungary; Clinical Cardiovascular Research Center, University of Rochester, Rochester, NY, USA; Department of Cardiology, Goethe University, University Hospital, Frankfurt, Germany; Breda and WCN, Amphia Ziekenhuis, Utrecht, Netherlands; Division of Cardiology, University of Ottawa Heart Institute, Ottawa, Ontario, Canada; Department of Cardiology, Oslo University Hospital Ulleval, Oslo, Norway; Institute of Clinical Medicine, University of Oslo, Oslo, Norway; Department of Cardiology, J.W. Goethe University, Frankfurt am Main, Germany; Heart, Vascular and Neurology Theme, Karolinska University Hospital, Stockholm, Sweden; Department of Cardiology, Karolinska Institutet, Karolinska University Hospital, Stockholm, Sweden; Department of Cardiology, The Institute for Clinical and Experimental Medicine (IKEM), Praha 4, Czech Republic; Arrhytmia Unit, Hospital Universitario La Luz, Madrid, Spain; Cardiology Clinical Academic Group Molecular & Clinical Sciences Institute, City St George's University of London, London, UK; Department of Cardiology, University Hospital Basel, Basel, Switzerland; Department of Cardiology, Cardiovascular Research Institute Basel, Basel, Switzerland; Department of Medicine, Medical University of South Carolina, Charleston, SC, USA; Department of Cardiology, Herlev-Gentofte Hospital, Copenhagen, Denmark; Population Health Research Institute, McMaster University, 237 Barton Street East, Hamilton, Ontario, L8L2X2, Canada

**Keywords:** Apixaban, Cost-effectiveness, Subclinical atrial fibrillation, Stroke prevention

## Abstract

**Aims:**

Apixaban was superior to aspirin for the prevention of stroke or systemic embolism in participants with subclinical atrial fibrillation (SCAF) in the Apixaban for the Reduction of Thromboembolism in Patients With Device-Detected Subclinical Atrial Fibrillation trial. This was especially true for those with CHA_2_DS_2_-VASc score > 4. Understanding the cost-effectiveness of treating SCAF is important for decision-makers.

**Methods and results:**

Canadian, UK, German, and US direct healthcare costs [in 2023 US dollars (USD)] were applied to hospitalized events (including strokes and bleeds) and study drugs for all participants with a CHA_2_DS_2_-VASc score > 4 to determine the mean cost per participant during the trial (mean follow-up 3.5 years). A daily cost of $0.63, $0.11, $2.26, and $6.06 for apixaban in Canada, the UK, Germany, and the USA was used. If in-trial results were not cost-saving (below $0), the prospective plan was to perform a lifetime cost-effectiveness analysis using a Markov model and a willingness-to-pay of 50 000 USD per quality-adjusted life year (QALY). After considering the cost of study medication and clinical events over 3.5 years, apixaban was dominant (cost-saving and more effective) in Canada (−$2301) and the UK (−$902) but cost more in Germany and the USA ($600 and $1990, respectively). Over a lifetime, treatment with apixaban produced a net gain of 0.107 QALYs, but with costs in both Germany ($2623 more) and the USA ($9110 more), yielding an incremental cost-effectiveness ratio of $24 514 per QALY for Germany and $85 140 for the USA.

**Conclusion:**

In patients with SCAF and a CHA_2_DS_2_-VASc score > 4, apixaban is cost saving in Canada and the UK and cost-effective in Germany. Apixaban was not cost-effective in the USA under the base cost assumption but would be cost-effective at a daily cost of $4.35 and cost saving at $3.59.

## Graphical Abstract

**Graphical Abstract:**
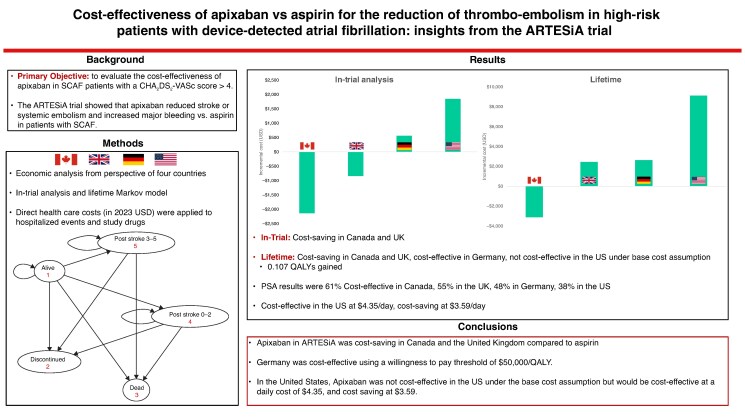

What’s new

- Apixaban in the Apixaban for the Reduction of Thromboembolism in Patients With Device-Detected Subclinical Atrial Fibrillation trial has been shown to reduce the risk of stroke in patients with subclinical atrial fibrillation especially those with CHA_2_DS_2_-VASc > 4, but the cost-effectiveness of this has not been evaluated in Canada, the UK, Germany, and the USA.
- Apixaban was cost saving in Canada and the UK during the trial period.
- At a cost-effectiveness threshold of 50 000 USD per quality-adjusted life year, apixaban was cost-effective over a lifetime in Germany, but not cost-effective in the USA.

## Introduction

Atrial fibrillation (AF) is the most common form of cardiac arrhythmia and significantly increases the risk of ischaemic stroke and systemic embolism, especially among the elderly.^1,2^ Due to the risk of stroke, patients are routinely anticoagulated with warfarin or a direct oral anti-coagulant (DOAC). Subclinical atrial fibrillation (SCAF), also known as device-detected AF (DDAF), is a common form of AF that is asymptomatic and detectible with long-term, continuous cardiac rhythm monitoring by an implanted cardiac pacemaker or defibrillator.^3^ Given the increased risk of bleeding associated with oral anticoagulation management of SCAF with oral anticoagulation is contentious.

The Apixaban for the Reduction of Thromboembolism in Patients With Device-Detected Subclinical Atrial Fibrillation (ARTESiA) trial demonstrated that apixaban was superior to aspirin in preventing stroke or systemic embolism among patients with DDAF, with the most pronounced benefit observed in those with a CHA_2_DS_2_-VASc score > 4.^4,5^ Given the cost of apixaban and the potential savings from fewer strokes, an evaluation of the cost-effectiveness is important to inform policy makers. We hypothesize that apixaban in patients with a CHA_2_DS_2_-VASc score > 4 will be cost-effective due to cost savings from stroke prevention.

## Methods

### Clinical trial

The details and results of the ARTESiA trial and the related subgroup analysis by CHA_2_DS_2_-VASc tertiles have been previously published.^4,5^ The overall clinical trial randomized 4012 participants from 16 countries between May 2015 and July 2021 to receive either apixaban 5 mg bid or ASA 81 mg od. Participants were eligible if they were at least 55 years with SCAF and a CHA_2_DS_2_-VASc score of at least 3. Participants 75 or older were also eligible if they had a history of stroke with no other risk factors. The trial found that apixaban 5 mg bid reduced the occurrence of the primary outcome of stroke or systemic embolism by 37% compared to ASA (HR 0.63; 95% CI: 0.45–0.88; *P* = 0.007). To identify groups within the trial that may benefit more from apixaban, the ARTESiA trial analysed prespecified subgroups. A subgroup analysis by CHA_2_DS_2_-VASc tertile (<4, =4, and >4) found that the largest risk reduction for stroke or systemic embolism was in participants with CHA_2_DS_2_-VAS > 4 (absolute risk reduction: −3.95; 95% CI: −6.72 to −1.19).^5^ In addition, a smaller subgroup of participants with subclinical AF and a previous history of stroke or transient ischaemic attack (TIA) had a lower risk of stroke or systemic embolism compared to those with no history but these results were not adjusted for multiplicity.^6^ Therefore, our base case analysis is limited to participants with a CHA_2_DS_2_-VASc score > 4, but an exploratory analysis for participants with a previous history of stroke or TIA was also performed.

### In-trial analysis design

An in-trial analysis from healthcare perspectives specific to Canada, the UK, Germany, and the USA was performed utilizing individual patient-level data collected from the trial. We hypothesized that apixaban in patients with a CHA_2_DS_2_-VASc score > 4 would be cost saving due to cost savings from stroke prevention. Costs for cardiovascular events, gastrointestinal (GI)/genitourinary (GU) bleeds, and study medications were included in this analysis. Each country’s unit costs were applied to all participants with a CHA_2_DS_2_-VASc > 4, giving a country-specific perspective using all participants in this subgroup. All hospitalized systemic emboli, strokes, myocardial infarctions (MIs), TIAs, heart failure (HF), and GI/GU bleeds were included with the frequency of these events derived from data pulled from the study case report forms (CRFs) (*Table 1*; Supplementary material online, *Table S1*). Progression to clinical AF/SCAF > 24 h occurred in almost 33% (32.5%) of participants with a CHA_2_DS_2_-VASc > 4.^7^ Event and study drug data after the date of this occurrence were not included in our analysis. Other events and bleeding sites were captured in the study CRFs but were not included in our analysis due to similar/low frequency between both groups, or their occurrence was not related to the study medication. If in-trial results were not cost saving, the prospective plan was to perform a lifetime cost-effectiveness analysis using a Markov model and assume a willingness to pay of 50 000 US dollars (USD) per quality-adjusted life year (QALY).

**Table 1:** Number of included events in the CHA₂DS₂-VASc > 4 subgroup for Canada^a^

	ASA	Apixaban
Systemic emboli	2	1
GI/GU bleed	9	28
MI	28	24
Non-disabling ischaemic stroke	13	5
Disabling ischaemic stroke	17	9
Fatal ischaemic stroke	1	3
Non-disabling haemorrhagic stroke	1	2
Disabling haemorrhagic stroke	3	0
Fatal haemorrhagic stroke	3	0
TIA	29	25
HF	105	124

ASA, acetylsalicylic acid; GI/GU, gastrointestinal/genitourinary; HF, heart failure; ICER, incremental cost-effectiveness ratio; MI, myocardial infarction; TIA, transient ischaemic attack.

^a^Data reorganized for the UK, Germany, and the USA available in the appendix.

Canadian non-stroke event unit costs were obtained from internal Hamilton Health Sciences data and have been used in previous publications.^8,9^ These costs were inflated to 2023 CAD and converted to US dollars (*Table 2*). The daily cost of apixaban was obtained from the Ontario Drug Benefit formulary (0.82 CAD = 0.63 USD). The cost of apixaban in the UK was based on the NHS daily tariff cost of apixaban (0.08 GBP = 0.11 USD) (see Supplementary material online, *Table S1*) obtained from the British National Formulary.^10^ Non-stroke event costs were based on weighted averages of relevant Healthcare Resource Group (HRG) codes for each event.^11^

**Table 2:** Unit costs used for the in-trial analysis—Canada^a^

	Canada
	Cost (USD)
Systemic emboli	11 609
GI/GU bleed	7356
MI	10 677
Non-disabling ischaemic stroke	50 882
Disabling ischaemic stroke	113 557
Fatal ischaemic stroke	32 109
Non-disabling haemorrhagic stroke	50 882
Disabling haemorrhagic stroke	113 557
Fatal haemorrhagic stroke	32 109
TIA	4023
HF	10 551

GI/GU, gastrointestinal/genitourinary; HF, heart failure; MI, myocardial infarction; TIA, transient ischaemic attack.

^a^Costs for the UK, Germany, and the USA available in the appendix.

In Germany, the daily cost of apixaban was obtained from personal communications (A. Benz, personal communication, 18 December 2024) (2.59 Eur = 2.26 USD) (see Supplementary material online, *Table S1*). Non-stroke event costs were from our previous work on the cost-effectiveness of rivaroxaban in the Cardiovascular Outcomes for People Using Anticoagulation Strategies (COMPASS) trial.^9^ For the US perspective analysis, the price of apixaban was assumed to be $6.06 per day. This was based on the price of rivaroxaban 2.5 mg bid as reported by the Institute for Clinical and Economic Review.^12^ We used this cost as a pragmatic cost of apixaban that included rebates and coupons was not readily available. The cost of events was based on 2021 Medicare data from MEDPAR and inflated to 2023 USD^13^(see Supplementary material online, *Table S1*).

The cost of strokes for all countries was obtained from sources different from those described above^14–17^. Unlike other events where costs are concentrated in the index hospitalization, the economic impact of strokes often lasts much longer. To reflect this reality, we use a 1-year cost of stroke obtained from literature for the in-trial analysis.

In this analysis, categorical data are reported as frequencies and continuous data as means. Totals are presented as mean costs per participant with 95% confidence intervals (CIs). To account for the fact that cost data are not normally distributed, the bias-corrected and accelerated method was used to bootstrap 5000 samples to determine 95% CIs. Analyses were performed with Stata 17 (StataCorp. 2017. Stata Statistical Software Release 17. College Station, Tx: Statacorp LLC).

### Lifetime cost-effectiveness

**Figure 1:**
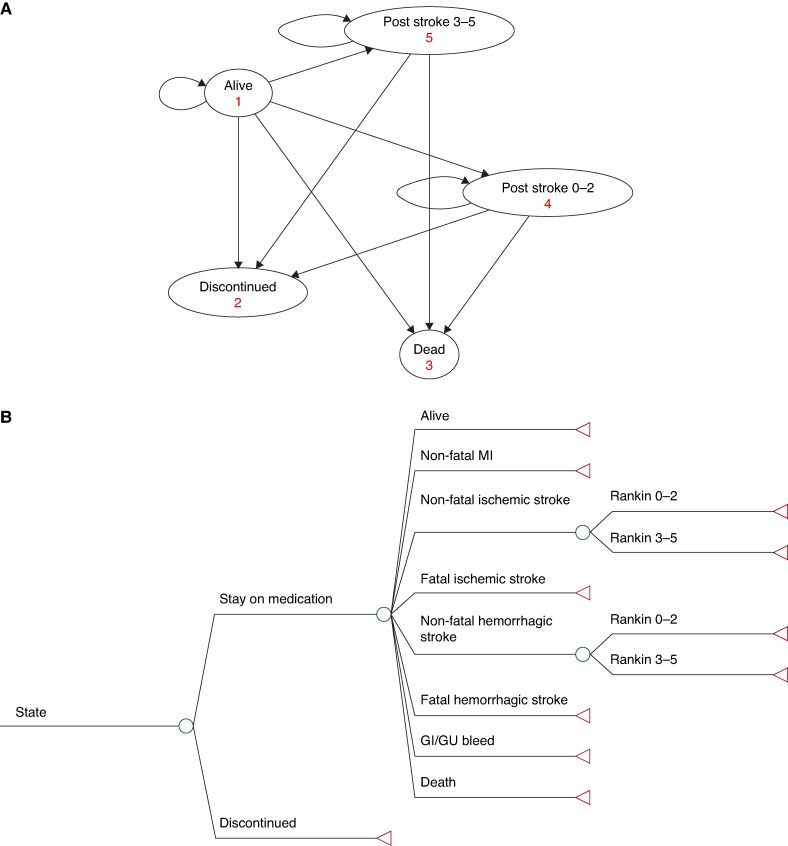
(*A*) Simplified state transition diagram. Participants begin in the alive state. Every cycle (3 months) they have a chance to move to another state or remain in the same state based on their path in (*B*). GI/GU, gastrointestinal/genitourinary; MI, myocardial Infarction. (*B*) Patient flow for States 1, 4, and 5.

A deterministic Markov model was created to simulate the total costs and QALYs if the ARTESiA trial had run for 23 years (*Figure 1A* and *B*). This time horizon was chosen to ensure all estimated costs and effects were captured.^18^ The model consisted of five states: alive, dead, discontinued, Rankin 0–2 post-stroke, and Rankin 3–5 post-stroke. It was assumed everyone began in the alive state with a mean age of 77 years and remained in the model for a maximum of 23 years. If discontinuation did not occur, one of seven events (MI, GI/GU bleed, fatal/non-fatal ischaemic stroke, fatal/non-fatal haemorrhagic stroke, and death), or no event, could be experienced. Patients who experience an MI or GI/GU bleed would begin the next cycle in the alive state.

Transition to one of the post-stroke states happens if a stroke was experienced during the alive state. Post-stroke severity was based on the distribution seen for the CHA_2_DS_2_-VASc > 4 subgroup. Once in a post-stroke state, participants had similar options as stroke naïve participants but with added long-term stroke costs and lower utility scores commensurate with the severity of the stroke. If a subsequent stroke were to occur, the severity would be limited to the severity of the first with the same associated costs and utilities.

Non-cardiac death probabilities were based on data from published US lifetables.^19^ One cycle in this model was equivalent to 3 months and included a half-cycle correction.

Daily drug costs in the Markov model were the same as the in-trial analysis. Costs for fatal and non-fatal MIs, GI/GU bleeds, and the index hospitalization for strokes were also the same as the in-trial analysis (see Supplementary material online, *Table S2*). Post-stroke costs were derived from published literature.^15,16^ Additional details can be found in the Supplementary material online, *Appendix*.

Transition probabilities were derived from event rates calculated using participant time to event data from the ARTESiA trial. Event data that occurred after the date of progression to clinical AF/SCAF > 24 h were not included. Annual event rates were converted to quarterly event rates and then converted to quarterly probabilities for use in the model (see Supplementary material online, *Table S3*).

As utilities were not collected as part of the ARTESiA trial, we used a utility of 0.87 based on data collected in the COMPASS trial. This utility value was used for event-free, GI/GU bleed, and non-fatal MIs (see Supplementary material online, *Table S3*). This utility value was also adjusted for stroke severity using published quality of life data for stroke patients.^20,21^ Additional details regarding this approach can be found in the Supplementary material online, *Appendix*. Utility values were assumed to be the same for both treatment groups. Although our utility scores use a US value set and are not unique to the countries in our analysis, we do not expect this to influence our conclusions for Germany since it has been shown that there is a very strong positive correlation (ρ = 0.97) between German and US value sets.^22^

All costs and QALYs were discounted at 3% per year.

### Sensitivity analyses

Deterministic sensitivity analyses were performed for both in-trial and modelled portions of our analysis. One-way sensitivity analyses alter the cost of strokes by ±50% and the cost of apixaban by ±90%. The daily cost of apixaban required to achieve no difference in cost between treatment groups in Germany and the USA was also determined.

For the modelled portion of our analysis, multiple one-way sensitivity analyses were conducted varying event probabilities by ±50% and study drug costs by ±90%. The results of these analyses were presented in a tornado diagram.

A probabilistic sensitivity analysis was also performed to explore parameter uncertainty in our model. This, plus a deterministic analysis, was also done for Canada and the UK to confirm the findings of our in-trial analysis. Event probabilities were simulated using a Dirichlet distribution, costs using a gamma distribution, and beta for discontinuation, utilities, and stroke severity distribution.

## Results

### In-trial

During the trial period, cost savings from fewer events were observed in all countries in our analysis. In Canada, savings attributable to events amounted to −$2804 (95% CI: −$5630–$22); in the UK, it was −$970 (95% CI: −$1980–$41) (*Table 3*). Event savings in Germany and the USA were −$1259 (95% CI: −$2455 to −$63) and −$2874 (95 CI: −$5945–$197), respectively. In all countries, these savings were largely a result of fewer disabling/severe ischaemic strokes in the apixaban arm.

**Table 3:** Mean in-trial costs per participant in each country (USD)

	Canada		UK		Germany		USA	
	ASA	Apixaban	ASA	Apixaban	ASA	Apixaban	ASA	Apixaban
Events	$8792	$5988	$3154	$2184	$3894	$2635	$9517	$6643
Study drug	$24	$527	$24	$92	$24	$1883	$24	$4888
Total	$8816	$6515	$3178	$2276	$3918	$4518	$9541	$11 531
Incremental cost	−$2301 (95% CI: −$5125–$523)		−$902 (95% CI: −$1912–$109)		$600 (95% CI: −$596–$1796)		$1990 (95% CI: −$1086–$5066)	
Incremental cost (discounted)	−$2138		−$838		$558		$1849	

ASA, acetylsalicylic acid; CI, confidence interval; USD, US dollars.

In Canada and the UK, these savings were sufficient to absorb the cost of apixaban during the trial where the mean drug cost per participant was $527 for apixaban compared to $24 for ASA in Canada and $92 for apixaban compared to $24 for ASA in the UK. The net result of this was that apixaban was cost saving in both Canada (−$2301; 95% CI: −$5125–$523) and the UK (−$902; 95% CI: −$1912–$109).

In Germany, the higher daily cost of apixaban ($2.26) resulted in a mean drug cost per participant of $1883 for apixaban compared to $24 for ASA. This exceeded cost savings from events and resulted in a mean total in Germany of $600 (95% CI: −$596–$1796). The highest apixaban costs in our study were from the USA where at $6.06 per day, the mean drug cost per participant was $4888 for apixaban vs. $24 for ASA, which also exceeded cost savings from events and resulted in a mean total of $1990 (95% CI: −$1086–$5066) in the USA.

### In-trial sensitivity analysis

In Canada and the UK, our findings were not sensitive to changes to the cost of strokes and apixaban; apixaban in ARTESiA remained cost saving under these conditions (*Table 4*). In Germany and the USA, the threshold cost (total mean difference in cost of $0) was $1.58/day (vs. $2.26) and $3.59/day (vs. $6.06), respectively.

**Table 4:** In-trial sensitivity analysis

	Canada	UK	Germany	USA
Base case	−$2301	−$902	$600	$1990
Stroke −50%	−$733	−$377	$1236	$3667
Stroke +50%	−$3869	−$1427	−$36	$314
Apixaban cost −90%	−$2775	−$2409	−$1147	−$2409
Apixaban cost +90%	−$1826	−$820	$2243	$6389

### Lifetime cost-effectiveness

As the in-trial economic results in Canada (−$2301) and the UK (−$902) were already cost saving and the ARTESiA trial has demonstrated the clinical benefits of apixaban, no lifetime cost-effectiveness analysis for the Canadian and UK perspective was necessary (dominant strategy). In Germany, the mean total cost per participant over a lifetime was $7560 for the apixaban group compared to $4937 for ASA, resulting in a mean difference of $2623. The total mean cost per participant over a lifetime in the USA was higher at $18 424 for the apixaban group compared to $9314 for ASA, resulting in a mean difference of $9110 (*Table 5*). Over this same time, horizon participants in the apixaban group accrued 4.995 QALYs compared to 4.888 for ASA. With incremental costs of $2623 and $9110 for Germany and the USA, respectively, incremental QALYs of 0.107, the incremental cost-effectiveness ratio (ICER) for Germany would be $24 514/QALY and $85 140/QALY for the USA.

**Table 5:** Lifetime mean total cost difference per patient in USD

	Canada		UK		Germany		USA	
	ASA	Apixaban	ASA	Apixaban	ASA	Apixaban	ASA	Apixaban
Costs	$9935	$6829	$5507	$3070	$4937	$7560	$9314	$18 424
Incremental cost	−$3106		−$2437		$2623		$9110	
Incremental cost (discounted)	−$2707		−$2129		$2319		$8032	
QALYs	4.888	4.995	4.888	4.995	4.888	4.995	4.888	4.995
Incremental QALYs	0.107		0.107		0.107		0.107	
Incremental QALYs (discounted)	0.086		0.086		0.086		0.086	
ICER	Dominant		Dominant		$24 514/QALY		$85 140/QALY	
ICER (discounted)	Dominant		Dominant		$26 965/QALY		$93 395/QALY	

ASA, acetylsalicylic acid; ICER, incremental cost-effectiveness ratio; QALY, quality-adjusted life year; USD, US dollars.

**Figure 2:**
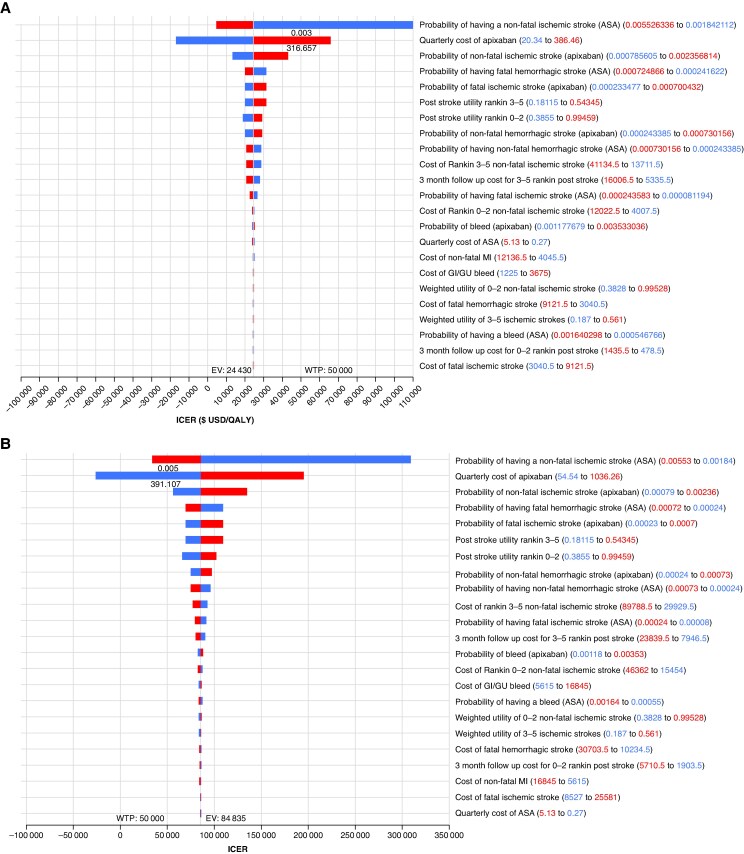
Tornado diagram of one-way deterministic sensitivity analyses for (*A*) Germany and (*B*) the USA. Bars represent the impact on the ICER when each input is varied above versus below its base case value. Bars corresponding to inputs higher than the base case are shown alongside inputs lower than the base case. ASA, acetylsalicylic acid; EV, expected value; GI/GU, gastrointestinal/genitourinary; ICER, incremental cost-effectiveness ratio; MI, myocardial infarction.

**Figure 3:**
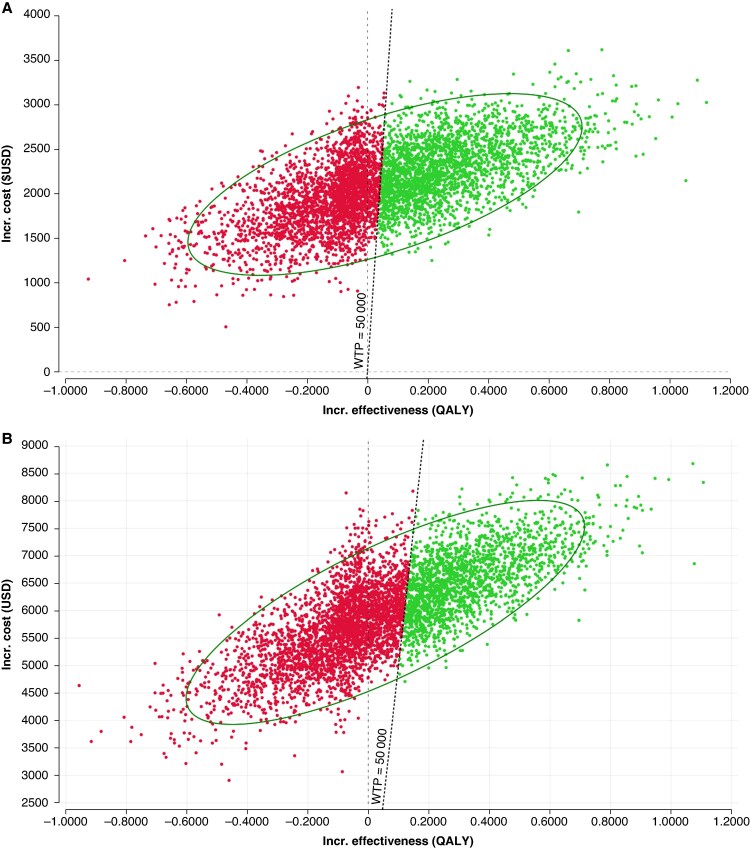
Probabilistic sensitivity analysis plot of 5000 samples for (*A*) Germany and (*B*) the USA. Points to the left of the dotted line represent samples that are above a willingness-to-pay threshold of 50 000 USD per QALY and therefore not cost-effective. Points to the right are cost-effective. QALY, quality-adjusted life year; USD, US dollars; WTP, willingness-to-pay.

In a series of one-way sensitivity analyses, all probabilities were varied by ±50% and all costs by ±90%. Our results were sensitive to changes in the probability of strokes in the ASA arm, as well as to the cost of apixaban similar to our in-trial sensitivity analysis (*Figure 2A* and *B*). In our probabilistic sensitivity analysis, apixaban was cost-effective in 48% of the samples for Germany and 38% in the USA with none of the samples being cost saving for either country (*Figure 3A* and *B*). In Canada, apixaban was 61% cost-effective with 100% of the samples being cost saving. In the UK, apixaban was 55% cost-effective with 85% of samples being cost saving (see Supplementary material online, *Figure S1*).

### History of stroke or transient ischaemic attack in-trial analysis

For ARTESiA participants with a previous history of stroke or TIA, apixaban was cost saving in all four countries considered in our analysis (see Supplementary material online, *Table S4*). In Canada, after adding the cost for study medication, the total average difference was −$4950 (95% CI −$10 897–$998) in favour of apixaban. In the UK, the total mean difference was −$1854 (95% CI: −$3912–$204). Apixaban saved −$759 (−$3101–$1584) per participant in Germany. Apixaban was also cost saving (−$1662; 95% CI: −$7830–$4507) after factoring in the cost of study medication.

## Discussion

A pre-planned subgroup analysis of ARTESiA trial demonstrated that among patients with SCAF and a CHA_2_DS_2_-VASc > 4, the rate of stroke or systemic embolism was 2.25%/year with aspirin compared to 0.98%/year with apixaban, the number needed to treat (NNT) to prevent stroke or systemic embolism was only 25 (95% CI: 15–84), and the risk–benefit ratio was in favour of treating these patients. Our analysis found that apixaban 5 mg bid was a dominant strategy (cost saving and clinically superior) in Canada and the UK. In Germany, while apixaban was associated with higher costs, the use of apixaban would be cost-effective with an ICER of $24 514. In the USA, the use of apixaban would not be cost-effective with an ICER $85 140/QALY. While cost savings from fewer events were present in each country in our analysis, the cost of apixaban was a larger influence. Our one-way sensitivity analyses confirmed this and demonstrated the robustness of our results to variations in stroke costs. Although our probabilistic sensitivity analysis results for Canada and the UK were 61% and 55% cost-effective, respectively, 100% of samples in Canada and 85% in the UK were cost saving and with the deterministic results support the findings of our in-trial analysis.

In our analysis, we use a cost-effectiveness threshold of 50 000 USD/QALY. However, cost-effectiveness thresholds vary from country to country and in some cases are only informally referenced. In Germany, no explicit threshold exists, but an ‘efficiency frontier analysis’ is performed based on all relevant comparator treatments, and an implied intervention-specific threshold is determined. Attempts at assigning a numerical value to this threshold have suggested it could be as low as 10 899 USD (€109 712 023) to as high as 47 461 USD.^23,24^ In the USA, no government-mandated threshold exists; however, the American College of Cardiology/American Heart Assocation (ACC/AHA) in the USA suggests that an ICER below $50 000/QALY is of high value.^25^

Oral anticoagulants prevent stroke but may cause major bleeding^26–28^. Secondary analyses of ARTESiA suggest that more strokes are prevented than major bleeds caused among patients with a CHA_2_DS_2_-VASc score > 4, those with prior cardiovascular disease, and those with a prior stroke or systemic embolism.^5,6,29^ However, it is overly simplistic to give strokes and major bleeds the same weight when assessing the net benefit of anticoagulation. In ARTESiA, nearly half of all strokes were fatal or disabling, while most of major bleeds were non-fatal and did not require procedural or surgical intervention.^4^ It is well accepted that strokes are significantly more likely than major bleeds to be associated with subsequent impairment in quality of life and mortality.^30^ Importantly, patient preference studies also show that patients with AF place greater value on stroke reduction than bleeding risk, while physicians are more averse to bleeding.^31,32^ Additionally, current clinical risk scores, such as HAS-BLED and DOAC, demonstrate only modest predictive ability, showing area under the curve values of 0.65 (95% CI: 0.55–0.70) and 0.62 (95% CI: 0.59–0.71), respectively, for major bleeding prediction, emphasizing the limitations in bleeding risk stratification tools used to assist with anticoagulation decisions.^33^ This economic analysis provides one more method of weighing stroke prevention against bleeding risk by demonstrating cost savings for using apixaban among patients with AF and a CHA_2_DS_2_-VASc score > 4 in Canada and the UK and a favourably cost-effectiveness in Germany.

### Limitations

There are certain limitations with the methodology used in our analysis. As a result of different stroke severity grouping between countries, stroke costs are tallied differently for each country. The lack of granularity in the groupings for Canada and the USA could result in higher costs being applied to lower Rankin strokes, but our one-way sensitivity analyses that varied the cost of stroke by ±50% did not alter our in-trial conclusions for Canada and the USA.

Our analysis focused on hospitalized GI and GU bleeds. This could result in a slight underestimate of the costs associated with bleeding; however, these sites were the most frequent bleed sites that occurred in the ARTESiA trial. In addition, some out-of-hospital costs associated with bleeds might not be included in our analysis.

As utilities were not captured as part of the ARTESiA trial, the baseline utility in our study was based on the baseline utility in the COMPASS trial. Patients in this trial were younger but sicker compared to ARTESiA patients, and on balance, this makes it a reasonable proxy for ARTESiA patients with a CHA_2_DS_2_-VASc > 4.

While the ICER in Germany is below our willingness to pay of $50 000/QALY, our probabilistic sensitivity analysis indicates it would be cost-effective in <50% of samples. This divergence from our deterministic model is likely due to this being an analysis of a subgroup where the lower frequency of events is a source of uncertainty in our PSA.

As the price of apixaban in the USA can vary depending on rebates, coupons, or direction of governance, we used the daily cost of rivaroxaban 2.5 bid obtained from a cost-effectiveness report published by the Institute for Clinical and Economic Review group.^12^ While this cost is for a different drug, both rivaroxaban and apixaban are factor Xa inhibitors and by including discounts, rebates, and concessions to wholesalers and distributors and patient assistance programmes, the price we use is a pragmatic proxy for the price of apixaban. Pricing for patients on Medicare is highly variable, but estimates are that the average monthly cost is currently in the range of $54, and at this price, the cost-effectiveness would be favourable.^34^ Litigations and political processes are currently ongoing in the USA, and a generic version of apixaban could likely be available soon.

## Conclusions

At the end of the trial, apixaban 5 mg bid in ARTESiA participants with SCAF and a CHA₂DS₂-VASc > 4 was cost saving in Canada and the UK compared to aspirin. In Germany, apixaban was associated with higher costs during the trial but was considered cost-effective over a lifetime using a willingness-to-pay threshold of $50 000/QALY; however, our probabilistic sensitivity analysis identified a high risk of uncertainty with these results. In the USA, apixaban was more costly during the trial and was not cost-effective at the current estimated price of apixaban. Apixaban was not cost-effective in the USA under the base cost assumption but would be cost-effective at a daily cost of $4.35 and cost saving at $3.59.

## Supplementary Material

euaf195_Supplementary_Data

## Data Availability

Individual patient data used for this analysis cannot be shared publicly for the privacy of individuals that participated in the study. Cost and probability data underlying this article are available in the article and in its online Supplementary material. Other data underlying this article will be shared on reasonable request to the corresponding author.
