# Supplementary material for: Cost-effectiveness of apixaban vs. aspirin for the reduction of thrombo-embolism in high-risk patients with device-detected atrial fibrillation: insights from the ARTESiA trial
Source: Europace. 2025 Aug 31;27(9):euaf195. doi: 10.1093/europace/euaf195 (PMC12448926; doi:10.1093/europace/euaf195)
Supplement: euaf195_Supplementary_Data [file euaf195_supplementary_data.docx]

Table S1: Unit costs for events, and their frequency, used in the in-trial analysis for the UK, Germany and the US

UK

|  | **Cost (USD)** | **ASA** | **Apixaban** |
| --- | --- | --- | --- |
|  |  |  |  |
| **Systemic Emboli** | 7148 | 2 | 1 |
| **GI/GU Bleed** | 2573 | 9 | 28 |
| **MI** | 2976 | 28 | 24 |
|  |  |  |  |
| **Rankin 0 Ischemic Stroke** | 6333 | 2 | 0 |
| **Rankin 1 Ischemic Stroke** | 7449 | 5 | 5 |
| **Rankin 2 Ischemic Stroke** | 8567 | 6 | 0 |
| **Rankin 3 Ischemic Stroke** | 33035 | 5 | 3 |
| **Rankin 4 Ischemic Stroke** | 44699 | 7 | 5 |
| **Rankin 5 Ischemic Stroke** | 53090 | 5 | 1 |
| **Rankin 6 Ischemic Stroke** | 6333 | 1 | 3 |
|  |  |  |  |
| **Rankin 0 Hemorrhagic Stroke** | 6333 | 1 | 1 |
| **Rankin 1 Hemorrhagic Stroke** | 7449 | 0 | 1 |
| **Rankin 2 Hemorrhagic Stroke** | 8567 | 0 | 0 |
| **Rankin 3 Hemorrhagic Stroke** | 33035 | 2 | 0 |
| **Rankin 4 Hemorrhagic Stroke** | 44699 | 0 | 0 |
| **Rankin 5 Hemorrhagic Stroke** | 53090 | 1 | 0 |
| **Rankin 6 Hemorrhagic Stroke** | 6333 | 3 | 0 |
|  |  |  |  |
| **TIA** | 6333 | 29 | 25 |
|  |  |  |  |
| **HF** | 3739 | 105 | 124 |

Germany

|  | **Cost (USD)** | **ASA** | **Apixaban** |
| --- | --- | --- | --- |
| **Systemic Emboli** | 7148 | 2 | 1 |
| **GI/GU Bleed** | 2450 | 9 | 28 |
| **MI** | 8091 | 28 | 24 |
|  |  |  |  |
| **Rankin 0 Ischemic Stroke** | 6081 | 2 | 0 |
| **Rankin 1 Ischemic Stroke** | 6081 | 5 | 5 |
| **Rankin 2 Ischemic Stroke** | 19479 | 6 | 0 |
| **Rankin 3 Ischemic Stroke** | 29646 | 5 | 3 |
| **Rankin 4 Ischemic Stroke** | 57748 | 7 | 5 |
| **Rankin 5 Ischemic Stroke** | 60679 | 5 | 1 |
| **Rankin 6 Ischemic Stroke** | 6081 | 1 | 3 |
|  |  |  |  |
| **Rankin 0 Hemorrhagic Stroke** | 6081 | 1 | 1 |
| **Rankin 1 Hemorrhagic Stroke** | 6081 | 0 | 1 |
| **Rankin 2 Hemorrhagic Stroke** | 19479 | 0 | 0 |
| **Rankin 3 Hemorrhagic Stroke** | 29646 | 2 | 0 |
| **Rankin 4 Hemorrhagic Stroke** | 57748 | 0 | 0 |
| **Rankin 5 Hemorrhagic Stroke** | 60679 | 1 | 0 |
| **Rankin 6 Hemorrhagic Stroke** | 6081 | 3 | 0 |
|  |  |  |  |
| **TIA** | 6081 | 29 | 25 |
| **HF** | 4479 | 105 | 124 |

US

| **US** | **Cost (USD)** | **ASA** | **Apixaban** |
| --- | --- | --- | --- |
| **Non-Fatal Systemic Emboli** | 7148 | **2** | **1** |
| **Fatal Systemic Emboli** | 12557 | **0** | **0** |
|  |  |  |  |
| **Non-Fatal GI/GU Bleed** | 11230 | **9** | **27** |
| **Fatal GI/GU Bleed** | 15936 | **0** | **1** |
|  |  |  |  |
| **Non-Fatal Mi** | 11230 | **27** | **19** |
| **Fatal Mi** | 16703 | **1** | **5** |
|  |  |  |  |
| **Minor Isch Stroke** | 46454 | **13** | **5** |
| **Moderate Isch Stroke** | 81780 | **5** | **3** |
| **Severe Isch Stroke** | 153516 | **12** | **6** |
| **Fatal Isch Stroke** | 24081 | **1** | **3** |
|  |  |  |  |
| **Minor Hem Stroke** | 35525 | **1** | **2** |
| **Moderate Hem Stroke** | 73723 | **2** | **0** |
| **Severe Hem Stroke** | 149164 | **1** | **0** |
| **Fatal Hem Stroke** | 27496 | **3** | **0** |
|  |  |  |  |
| **TIA** | 6246 | **29** | **25** |
| **HF** | 11465 | **105** | **124** |

Table S2: Costs (USD) Used in Markov Model for Germany and the US

| **Description** | **German Costs (USD)** | **US Cost s (USD)** |
| --- | --- | --- |
| 3 month follow up cost for 0-2 Rankin Post Stroke | 957 | 3807 |
| 3 month follow up cost for 3-5 Rankin Post Stroke | 10671 | 15893 |
| Quarterly cost of apixaban | 203.4 | 545.4 |
| Quarterly cost of ASA | 2.7 | 2.7 |
| Cost of Fatal Hemorrhagic Stroke | 6081 | 20469 |
| Cost of Fatal Ischemic Stroke | 6081 | 17054 |
| Cost of Fatal MI | 8091 | 16703 |
| Cost of GI/GU Bleed | 2450 | 11230 |
| Cost of Rankin 0-2 Non-Fatal Hemorrhagic Stroke | 7995 | 22771 |
| Cost of Rankin 3-5 Non-Fatal Hemorrhagic Stroke | 27423 | 52675 |
| Cost of Rankin 0-2 Non-Fatal Ischemic Stroke | 7995 | 30908 |
| Cost of Rankin 3-5 Non-Fatal Ischemic Stroke | 27423 | 59859 |
| Cost of Non-Fatal MI | 8091 | 11230 |

Table S3: Quarterly Probabilities and utilities used in Deterministic Model

| Probability of Apixaban Discontinuation | 0.0233 |
| --- | --- |
| Probability of bleed (apixaban) | 0.0023553570 |
| Probability of non-CV death (apixaban) | Life Tables |
| Probability of fatal hemorrhagic stroke (apixaban) | 0 |
| Probability of fatal ischemic stroke (apixaban) | 0.0004669547 |
| Probability of non-fatal hemorrhagic stroke (apixaban) | 0.0004867707 |
| Probability of non-fatal ischemic stroke (apixaban) | 0.0015712094 |
| -Proportion of rankin 0-2 stroke with apixaban | 0.4375 |
| -Proportion of Rankin 3-5 stroke with Apixaban | 0.5625 |
| probability of non-fatal MI (apixaban) | 0.0016887537 |
| Probability of no event (apixaban) | 1-sum of all other probabilities |
|  |  |
| Probability of ASA discontinuation | 0.0234 |
| Probability of having a bleed (ASA) | 0.0010935317 |
| Probability of non-CV death (ASA) | Life Tables |
| Probability of having fatal hemorrhagic stroke (ASA) | 0.0004832440 |
| Probability of having fatal ischemic stroke (ASA) | 0.0001623886 |
| Probability of having non-fatal hemorrhagic stroke (ASA) | 0.0004867707 |
| Probability of having a non-fatal ischemic stroke (ASA) | 0.0036842240 |
| -Proportion of Rankin 0-2 stroke with ASA | 0.4118 |
| -Proportion of Rankin 3-5 stroke with ASA | 0.5882 |
| Probability having non-fatal MI (ASA) | 0.0022084493 |
| Probability of no event (ASA) | 1-sum of all other probabilities |
|  |  |
| Weighted utility of 0-2 non-fatal hemorrhagic stroke | 0.8129 |
| Weighted utility of 0-2 non-fatal ischemic stroke | 0.7656 |
| Post Stroke Utility Rankin 0-2 | 0.7710 |
| weighted utility of 3-5 hemorrhagic stroke | 0.2803 |
| weighted utility of 3-5 ischemic strokes | 0.3740 |
| Post Stroke Utility Rankin 3-5 | 0.3623 |
| utility for all other states | 0.87 |

Table S4: Mean in-trial costs for ARTESiA participants with previous stroke or transient ischemic attack (USD)

|  | **Canada** | | **United Kingdom** | | **Germany** | | **United States** | |
| --- | --- | --- | --- | --- | --- | --- | --- | --- |
|  | **ASA**  **N=174** | **Apixaban**  **N=172** | **ASA**  **N=174** | **Apixaban**  **N=172** | **ASA**  **N=174** | **Apixaban**  **N=172** | **ASA**  **N=174** | **Apixaban**  **N=172** |
| Events | $11310 | $5885 | $3946 | $2030 | $4928 | $2409 | $12215 | $6050 |
| Study Drug | $26 | $501 | $26 | $87 | $26 | $1786 | $26 | $4529 |
| Total | $11336 | $6386 | $3971 | $2117 | $4954 | $4195 | $12241 | $10579 |
| Incremental Cost | -$4950  (-$10897 to $998) | | -$1854  (-$3912 to $204) | | -$759  (-$3101 to $1584) | | -$1662  (-$7830 to $4507) | |

Figure S1: Probabilistic sensitivity analysis plot of 5000 samples for a) Canada and b) the UK

Legend: Points in red represent samples that are above a willingness-to-pay threshold of $50,000 USD per QALY and therefore not cost-effective. Points in green are cost-effective. WTP = Willingness-to-pay; USD = US Dollars; QALY = Quality Adjusted Life Years

a)


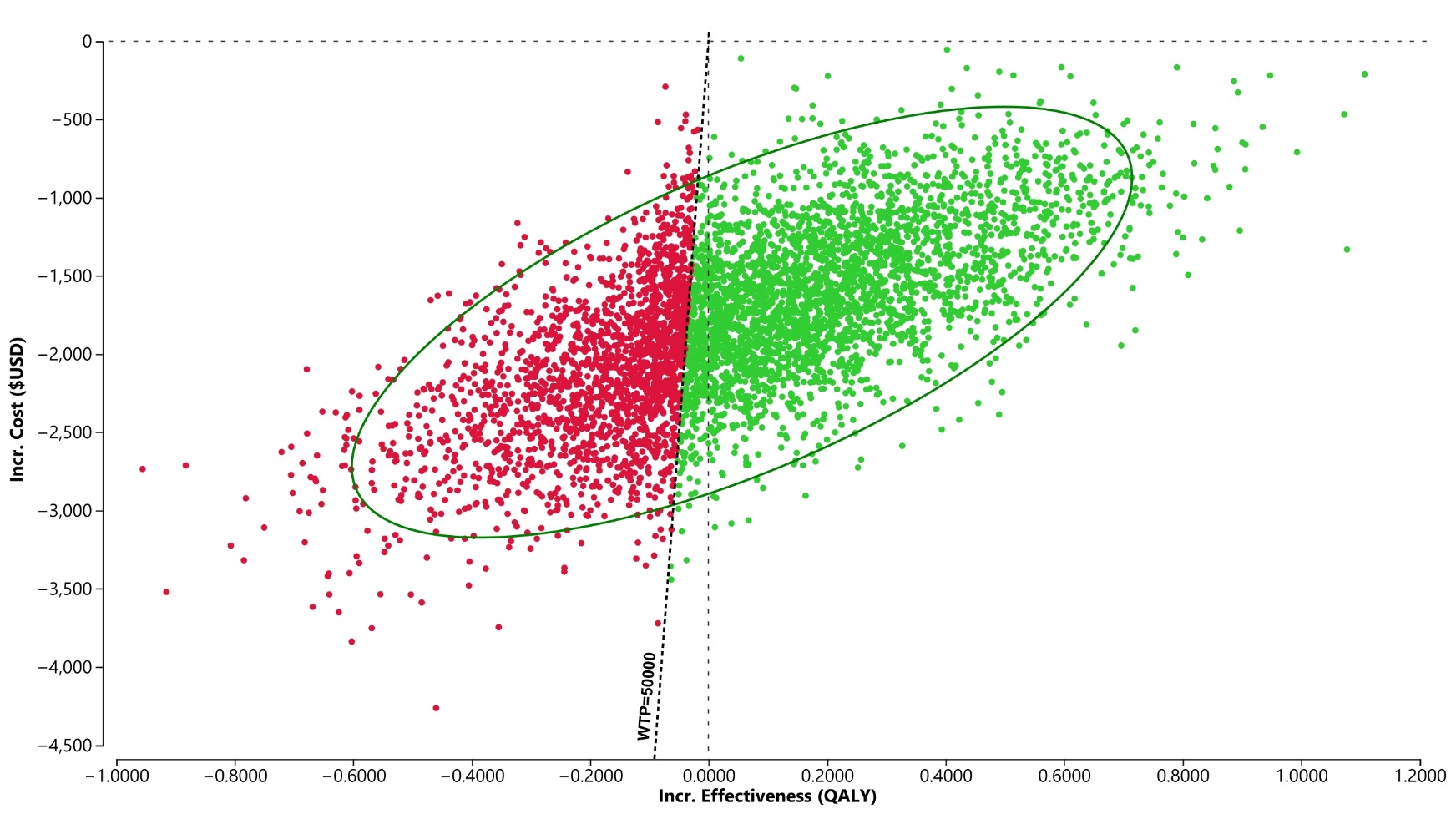


b)
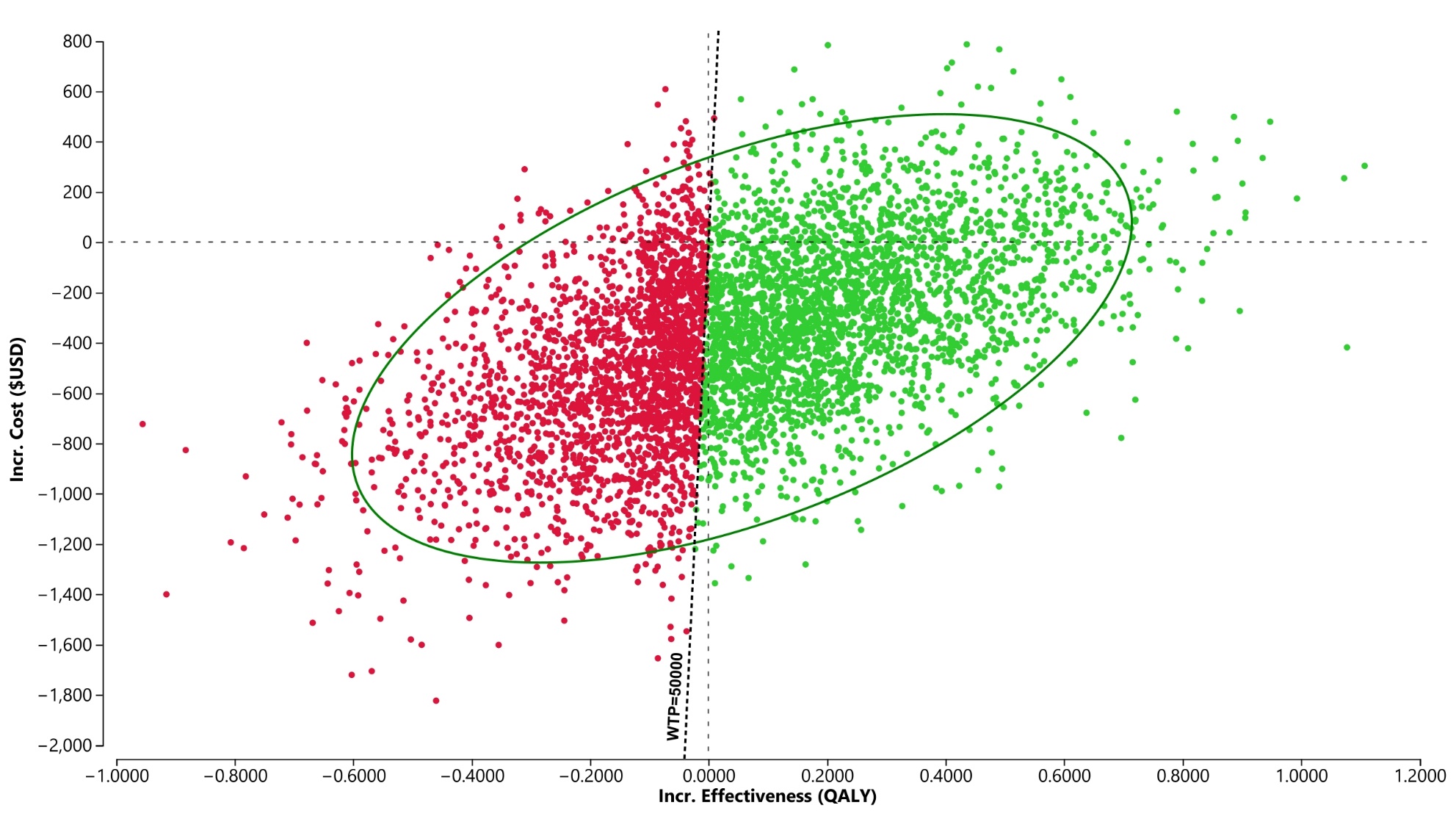


**Lifetime Cost-Effectiveness – Post Stroke Costs**

In Germany, post-stroke costs were based on annual long-term costs reported in a cost-effectiveness analysis of magnetic resonance imaging-guided thrombolysis in stroke patients ^1^. These costs, which were stratified by modified Rankin score, were then used to create weighted (based on CHA₂DS₂-VASc score >4 ARTESiA participant data) average costs for modified Rankin 0-2 and Rankin 3-5 annual post stroke costs and then converted to quarterly costs. Post-stroke costs were applied for every event-free cycle a participant experienced in the post-stroke state.

In the US, post-stroke costs were based on work by Simpson et al who developed stroke follow up costs in their cost-effectiveness analysis of the Interventional Management of Stroke – III trial ^2^. These costs were then adjusted in a similar manner to Germany’s post-stroke cost.

**Lifetime Cost-Effectiveness – Utilities**

Utilities were not collected as part of the ARTESiA trial so a utility of 0.87 based on data collected in the COMPASS trial was used. The COMPASS trial evaluated rivaroxaban plus aspirin versus aspirin alone for the prevention of cardiovascular events in participants with stable atherosclerotic disease. Although participants in this trial were younger than those in ARTESiA, they were also in poorer health. This utility value was used for event-free, GI/GU bleed and non-fatal MIs. Ischemic and hemorrhagic stroke utilities were obtained from two American studies that reported utilities stratified by Rankin score. These utilities were then averaged for each Rankin score from 0-5. For each increase in Rankin score, the corresponding percent decrease in utility was calculated. These percentages were then used to extrapolate the incremental decrease to our baseline utility of 0.87 for each Rankin score.

1. Muntendorf L-K, Konnopka A, König H-H, Boutitie F, Ebinger M, Endres M, Fiebach JB, Thijs V, Lemmens R, Muir KW, Nighoghossian N, Pedraza S, Simonsen CZ, Gerloff C, Thomalla G. Cost-Effectiveness of Magnetic Resonance Imaging-Guided Thrombolysis for Patients With Stroke With Unknown Time of Onset. *Value Health*. 2021;24:1620–1627.

2. Simpson KN, Simpson AN, Mauldin PD, Palesch YY, Yeatts SD, Kleindorfer D, Tomsick TA, Foster LD, Demchuk AM, Khatri P, Hill MD, Jauch EC, Jovin TG, Yan B, von Kummer R, Molina CA, Goyal M, Schonewille WJ, Mazighi M, Engelter ST, Anderson C, Spilker J, Carrozzella J, Ryckborst KJ, Janis LS, Broderick JP, Interventional Management of Stroke (IMS) III Investigators. Observed Cost and Variations in Short Term Cost-Effectiveness of Therapy for Ischemic Stroke in Interventional Management of Stroke (IMS) III. *J Am Heart Assoc*. 2017;6:e004513.
